# Myocarditis with preserved left ventricular ejection fraction in-hospital convalescent phase: insights from CMR and myocardial strain analysis

**DOI:** 10.3389/fcvm.2025.1613394

**Published:** 2026-01-12

**Authors:** Mengting Huang, Kailu Zhang, Guozhu Shao, Qinyue Luo, Yuting Zheng, Xiaoqing Liu, Yukun Cao, Heshui Shi

**Affiliations:** 1 Department of Radiology, Union Hospital, Tongji Medical College, Huazhong University of Science and Technology, Wuhan, China; 2 Hubei Provincial Clinical Research Center for Precision Radiology & Interventional Medicine, Wuhan, China; 3 Hubei Province Key Laboratory of Molecular Imaging, Wuhan, China

**Keywords:** cardiovascular diseases, MRI, myocarditis, prognosis, strain

## Abstract

**Background:**

The convalescent phase of myocarditis in patients with preserved left ventricular ejection fraction remains poorly characterized. Persistent myocardial inflammation may elevate the risk of major adverse cardiovascular events (MACE). Conventional assessment of left ventricular function is limited in sensitivity, whereas myocardial strain analysis provides detection of subclinical myocardial dysfunction.

**Methods:**

In this retrospective study, 317 patients with myocarditis underwent cardiac magnetic resonance (CMR). Feature-tracking CMR was used to quantify ventricular and atrial strain parameters. Late gadolinium enhancement (LGE) was assessed for myocardial tissue characterization. Patients were followed clinically, with MACE as the primary endpoint.

**Results:**

Among 146 patients included in the final analysis (71 male; mean age 26 ± 16 years), 36 experienced MACE. LGE was present in 54.8% of patients. In LGE positive patients, one, two, and three or more segments were involved in 6.25%, 16.25%, and 77.50% of cases, respectively. In univariable and multivariable Cox regression analyses, left ventricular global radial strain (*p* = 0.026) and left ventricular diastolic middle circumferential strain rate (*p* = 0.014) emerged as independent predictors of MACE.

**Conclusions:**

Left ventricular global radial strain may reflect persistent myocardial inflammation during convalescence from myocarditis. Myocardial strain parameters provide incremental prognostic value beyond conventional functional measures.

## 1 Introduction

Myocarditis, an inflammatory disease of the myocardium, is associated with substantial morbidity and mortality (1), making it a focus of extensive research. While the acute and sub-acute phases of the disease have been well studied (2, 3), the in-hospital convalescent phase, particularly in patients with preserved left ventricular ejection fraction (pLVEF), remains inadequately characterized. Current management during this phase is typically limited to supportive care, as standardized follow-up protocols are lacking (4). Nonetheless, residual myocardial inflammation may persist (5), potentially increasing the risk of major adverse cardiovascular events (MACE). Identification of prognostic factors at this stage is therefore critical for optimizing clinical management and improving long-term outcomes.

Left ventricular ejection fraction (LVEF) serves as the conventional metric for assessing systolic function but has limited sensitivity in detecting subtle myocardial impairment (6, 7). In contrast, myocardial strain provides a more comprehensive evaluation of myocardial deformation (8, 9) and can detect early, mild functional abnormalities that may be overlooked by LVEF. Recently, feature-tracking strain analysis has emerged as a valuable tool for assessing myocardial mechanics (10, 11).

Important knowledge gaps remain regarding the application of myocardial strain during recovery from myocarditis. The optimal strain parameters for risk stratification are still uncertain, and the prognostic value of strain has not been comprehensively validated in large cohorts (12). Furthermore, the relationship between myocardial strain and other biomarkers or imaging findings in this specific patient population has not been fully elucidated.

This study aims to investigate the clinical and cardiac magnetic resonance (CMR) characteristics, as well as the prognosis, of patients with pLVEF during the in-hospital convalescent phase of myocarditis. Our primary objective is to evaluate the prognostic value of late gadolinium enhancement (LGE) and myocardial strain parameters for predicting MACE in this population. We seek to identify risk factors associated with MACE and to determine the prognostic significance of myocardial strain, thereby contributing to improved clinical management and long-term outcomes in these patients.

## 2 Materials and methods

This retrospective study was approved by the institutional ethics committee, and the requirement for written informed consent was waived.

### 2.1 Study population

We conducted a retrospective analysis of 317 consecutive adult patients diagnosed with myocarditis at our institution between January 2015 and May 2022. All patients were managed and discharged in accordance with the 2013 European Society of Cardiology guidelines (13). The diagnosis of myocarditis was established based on clinical presentation, elevated cardiac biomarkers, electrocardiographic abnormalities, and CMR findings consistent with myocardial inflammation. Although endomyocardial biopsy represents the diagnostic gold standard, it was not routinely performed in this cohort due to its invasive nature and the presence of characteristic clinical and CMR profiles.

**Figure 1:**
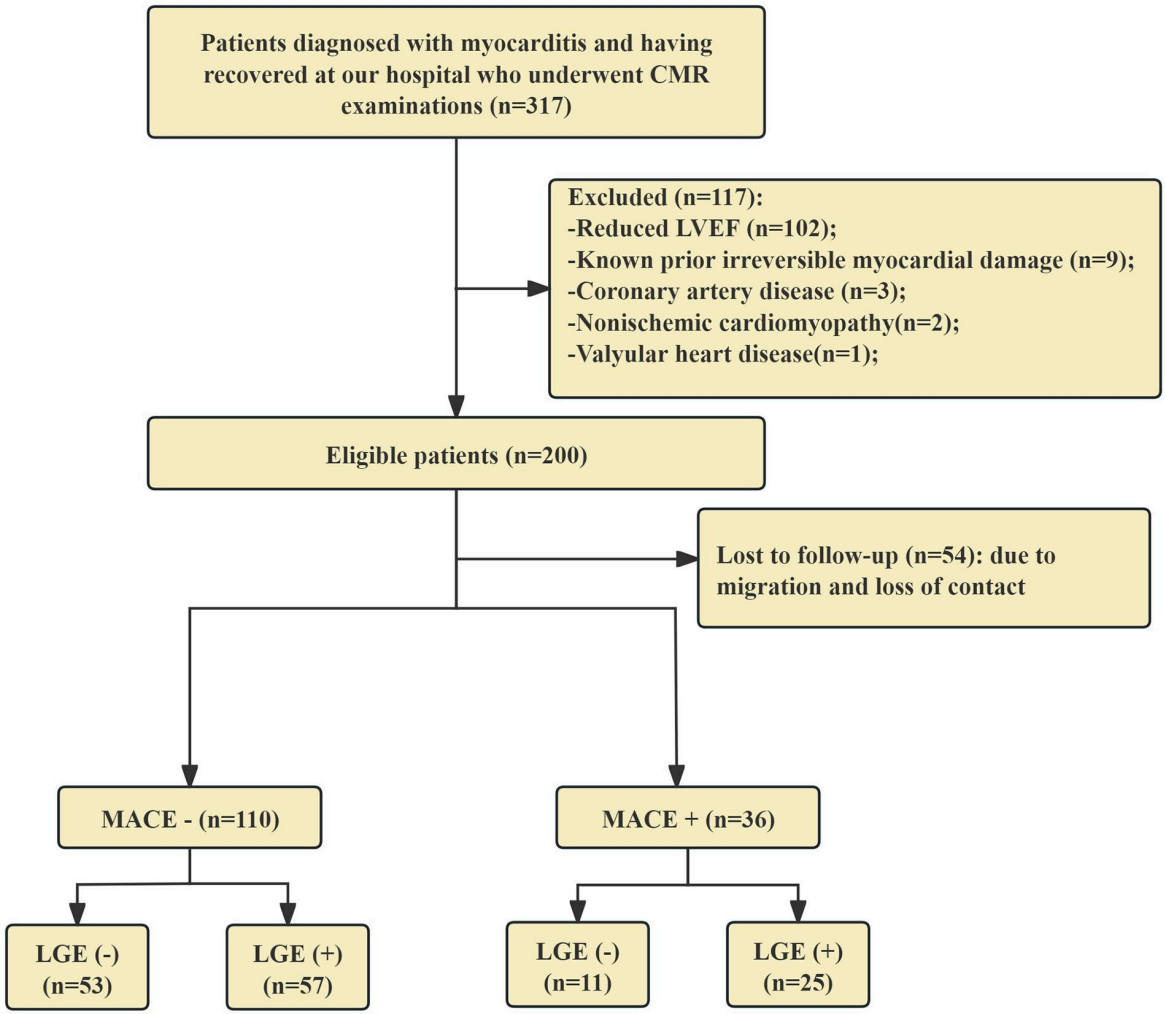
Patient flowchart. CMR, cardiovascular MRI; MACE, major adverse cardiovascular event; LGE, late gadolinium enhancement.

Exclusion criteria were as follows: (1) reduced LVEF (LVEF < 50%; *n* = 102); (2) history of myocardial infarction or pre-existing irreversible myocardial damage (*n* = 9); (3) coronary artery disease (*n* = 3); (4) non-ischemic cardiomyopathy (*n* = 2); (5) valvular heart disease (*n* = 1); and (6) congenital heart disease or suboptimal CMR image quality (*n* = 0). All patients received standard guideline-based supportive care for myocarditis; any immunosuppressive therapies were administered during the acute phase prior to convalescence. Baseline demographic and clinical data were collected during the in-hospital convalescent phase. A participant flow diagram is provided in Figure 1.

### 2.2 CMR protocols

CMR imaging was performed using either a 1.5 Tesla (Siemens Healthcare, Germany, Area) or a 3.0 Tesla scanner (Siemens Healthcare, Germany, Skyra), both equipped with vector-electrocardiographic gating and a phased-array surface coil. All acquisitions were obtained with patients in the supine position. Cine imaging was performed using a breath-hold steady-state free precession sequence in standard cardiac orientations, including short-axis, two-chamber, three-chamber, and four-chamber views. Contiguous short-axis slices covered the left ventricle from the atrioventricular ring to the cardiac apex. LGE images were acquired 10–15 min after intravenous administration of 0.2 mmoL/kg of a gadolinium-based contrast agent using a breath-hold phase-sensitive inversion-recovery sequence, in the same planes as the cine images. Detailed scanning parameters are provided in Supplementary Material S1.

All CMR scans were conducted on the day prior to hospital discharge, provided the patient had achieved clinical stability-defined as resolution of acute symptoms-and biochemical improvement (troponin normalization to ≤0.04 ng/mL). Scans were performed at a median of 18 days (interquartile range: 14–25 days) from the index event.

### 2.3 CMR image processing

All CMR analyses were performed using dedicated commercial post-processing software (CVI42, Circle Cardiovascular Imaging Inc., Calgary, Canada). Image analysis was conducted by two radiologists with 10 and 5 years of experience in CMR, respectively. On consecutive short-axis cine images, left ventricular (LV) and right ventricular (RV) endocardial and epicardial borders were automatically traced and then manually adjusted at end-systole and end-diastole. Global strain for the LV and RV was computed based on end-diastolic phase data. In basal slices, the outflow tract was included in the ventricular volume.

**Figure 2:**
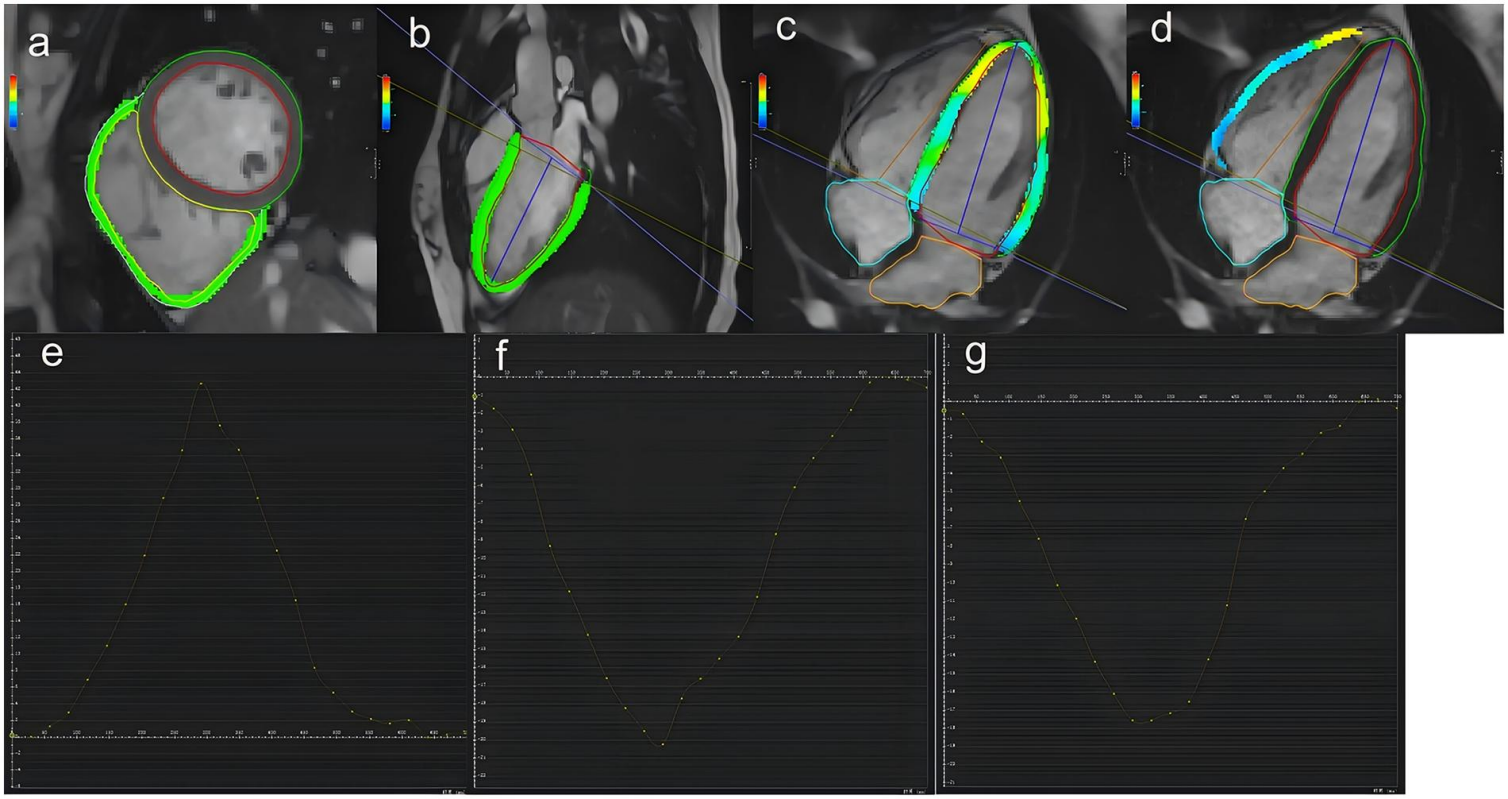
A schematic representation of left ventricular 3D peak global strain analysis. Strain analyses were performed on a stack of short-axis views **(a)** and a 3-chamber view **(b)** and 4-chamber view **(c,d)**. The curve of 3D peak global radial **(e)**, circumferential **(f)**, and longitudinal strain **(g)** of Left ventricular. The strain parameters are absolute values.

Left atrial (LA) volume was measured at ventricular end-systole from two- and four-chamber cine views, excluding the pulmonary veins and left atrial appendage. LA volume was calculated using the biplane area-length method: [0.85*(two-chamber LA area) *(four-chamber LA area)]/L, where L is the shortest distance from the posterior wall to the line connecting the mitral valve hinge points in both chambers. Right atrial (RA) area was measured at ventricular end-systole in the four-chamber view. The measurement methodology is illustrated in Figure 2.

**Figure 3:**
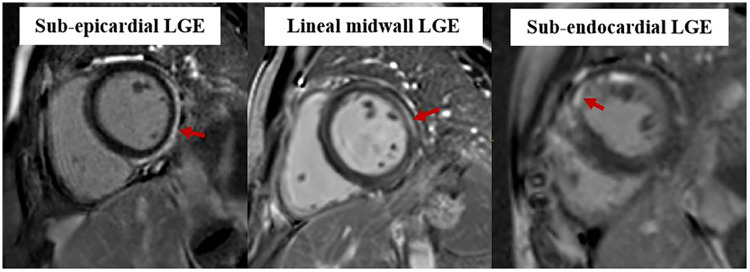
Examples of LGE patterns in patients recovering from myocarditis. LGE, late gadolinium enhancement.

All LGE images were independently evaluated by each radiologist to identify non-ischemic myocardial injury patterns, categorized as subendocardial/transmural, mid-myocardial, or sub-epicardial. Discrepancies were resolved by consensus with a senior radiologist (25 years of CMR experience). Representative LGE examples are provided in Figure 3.

### 2.4 Intra- and inter-observer variability analysis

To assess measurement reproducibility, thirty patients were randomly selected. Intra-observer variability was evaluated by one observer repeating measurements at two time points four weeks apart. Inter-observer variability was assessed by a second independent observer. Intraclass correlation coefficients (ICC) were calculated to quantify agreement, with ICC values interpreted as follows: below 0.75 indicated poor agreement; 0.75–0.90 indicated good agreement; and above 0.90 indicated excellent agreement. Reproducibility results for all key parameters are presented in Supplementary Material S2. Both intra- and inter-observer reproducibility were excellent for conventional biventricular volumetric and functional parameters.

### 2.5 Outcomes during follow-up

Clinical follow-up began at the time of CMR examination and was conducted via telephone interviews by a radiologist. The primary endpoint was MACE, a composite of cardiac death, cardiogenic shock, heart failure requiring hospitalization, and rehospitalization for recurrent myocarditis. All events were independently adjudicated by two cardiologists blinded to CMR and strain data. Disagreements were resolved by consensus with a third senior cardiologist.

During follow-up, 54 patients were lost (primarily due to relocation or loss of contact) and were excluded from outcome analyses, resulting in 146 patients included in the final analysis. A comparison of baseline characteristics between included patients and those lost to follow-up is provided in Supplementary Material S3. No statistically significant differences (all *p* > 0.05) were observed in any baseline characteristics.

### 2.6 Statistical analysis

Statistical analyses were performed using R software (version 4.4.3). Continuous variables with a normal distribution are presented as mean ± standard deviation and compared using the independent t-test. Non-normally distributed continuous variables are summarized as median with interquartile range and compared using the Mann–Whitney U test. Categorical variables are expressed as frequencies and percentages, and comparisons were made using the chi-square test or Fisher's exact test, as appropriate. Correlation analyses were performed using Pearson's correlation coefficient for normally distributed variables and Spearman's correlation for non-normally distributed variables. Optimal cutoff values for strain parameters were determined by receiver operating characteristic (ROC) curve analysis. Univariable and multivariable Cox proportional hazards regression models were used to identify independent prognostic factors associated with MACE. Survival analysis was conducted using the Kaplan–Meier method to evaluate the association between myocardial strain cutoff values and the incidence of adverse clinical outcomes. All statistical tests were two-sided, with statistical significance set at *p* < 0.05.

## 3 Results

### 3.1 Comparison of baseline characteristics between patients

A total of 146 patients (mean age 26 ± 16 years) were included in the final analysis. Baseline demographic and clinical characteristics are summarized in Table 1. The cohort consisted predominantly of young adults, with 8.2% reporting a smoking history and 3.4% reporting alcohol consumption. No statistically significant differences were observed between the MACE+ and MACE− groups regarding demographic characteristics, cardiovascular risk factors, or laboratory parameters. Among the 146 patients, 10 (6.8%) exhibited abnormalities in the mitral annular early-to-late diastolic velocity ratio or the mitral inflow early-to-late diastolic velocity ratio, suggestive of diastolic dysfunction. Regional wall motion abnormalities were identified by echocardiography in 4 patients (2.7%), including 3 (8.3%) in the MACE+ group and 1 (0.9%) in the MACE− group. Detailed transthoracic echocardiography measurements are provided in Supplementary Material S4.

**Table 1:** Baseline characteristics of the patients.

Characteristics	All patients (*n* = 146)	MACE− (*n* = 110)	MACE+ (*n* = 36)	*p* value
Demographics	–	–	–	–
Age (year)	26 ± 16	24 ± 16	29 ± 18	0.117
Gender (Male, %)	71 (49)	58 (53)	13 (36)	0.083
Body mass index	21.2 ± 4.0	21.0 ± 4.1	21.9 ± 3.7	0.244
Cardiovascular risk factors	–	–	–	–
Smoking (Yes, %)	12 (8.2)	10 (9.0)	2 (5.6)	0.503
Drinking (Yes, %)	5 (3.4)	3 (2.7)	2 (5.6)	0.418
Hypertension (Yes, %)	11 (7.5)	8 (7.3)	3 (8.3)	0.834
Diabetes (Yes, %)	1 (0.7)	0 (0)	1 (2.8)	0.079
Dyslipidemia (Yes, %)	2 (1.4)	1 (0.1)	1 (2.8)	0.402
Laboratory results	–	–	–	–
SBP (mmHg)	117 ± 16	116 ± 16	120 ± 15	0.188
DBP (mmHg)	72 ± 11	72 ± 11	71 ± 13	0.652
Hematocrit (%)	39.2 ± 4.0	39.1 ± 3.9	39.4 ± 4.3	0.697
WBC (10^9^/L)	6.9 ± 2.1	6.9 ± 1.9	7.0 ± 2.7	0.807
GFR (mL/min)	131.2 ± 27.7	132.7 ± 29.1	126.9 ± 23.3	0.279
CRP (mg/L)	16.5 ± 64.0	18.7 ± 73.1	9.5 ± 15.4	0.456
Hemoglobin (g/L)	130.4 ± 14.6	130.2 ± 13.9	130.9 ± 16.6	0.803
CK-MB (U/L)	25.0 ± 31.4	26.4 ± 32.6	17.9 ± 29.1	0.166
Albumin (g/L)	41.1 ± 4.3	40.9 ± 4.3	42.1 ± 4.0	0.142
Medication use (*n*, %)	–	–	–	–
β-blocker	59 (40.4)	44 (40.0)	15 (41.7)	0.860
ACE inhibitor or ARBs	105 (71.9)	80 (72.7)	25 (69.4)	0.704
Steroids	48 (32.9)	34 (30.9)	14 (38.9)	0.376
Intravenous immunoglobulin	28 (19.2)	22 (20.0)	6 (16.7)	0.659
Inotropes	135 (92.5)	103 (93.6)	32 (88.9)	0.349
ECG (*n*, %)	–	–	–	–
Sinus rhythm	67 (45.9)	50 (45.5)	17 (47.2)	0.853
Sinus tachycardia	22 (15.1)	18 (16.4)	4 (11.1)	0.444
Sinus bradycardia	13 (8.9)	10 (9.1)	3 (8.3)	0.890
ST-changes	19 (13.0)	13 (11.8)	6 (16.7)	0.453
Pathologic Q wave	3 (2.1)	1 (0.9)	2 (5.6)	0.088
Ventricular arrhythmia	10 (6.8)	7 (6.4)	3 (8.3)	0.685
Left bundle branch block	3 (2.1)	3 (2.7)	0 (0.0)	0.317
Atrial Premature	8 (5.5)	8 (7.3)	0 (0.0)	0.096
Atrioventricular block	1 (0.7)	0 (0)	1 (2.8)	0.079
TTE	–	–	–	–
Ejection fractions (%)	64.0 ± 6.0	63.6 ± 6.2	65.2 ± 5.5	0.162
Regional wall motion abnormalities (*n*, %)	4 (2.7)	1 (0.9)	3 (8.3)	0.018

MACE, major adverse cardiac events; LVEF, left ventricular ejection fraction; SBP, systolic blood pressure; DBP, diastolic blood pressure; BNP, brain natriuretic peptide; WBC, white blood cell; GFR, glomerular filtration rate; CRP, C-reactive protein; CK-MB, creatine kinase isoenzymes; ACE indicates angiotensin-converting enzyme; ARB, angiotensin II receptor blocker; ECG, electrocardiogram; TTE, transthoracic echocardiography.

CMR-derived ventricular and atrial parameters, both absolute and indexed to body surface area (BSA), are presented in Table 2 for the overall cohort and stratified by MACE status. Analysis of LV parameters showed that most volumetric and mass measurements were comparable between groups. No significant differences were observed in LV end-diastolic volume (EDV), end-systolic volume (ESV), stroke volume (SV), or mass, whether expressed as absolute or indexed values (all *p* > 0.05). However, LVEF was significantly lower in the MACE+ group compared to the MACE− group (*p* = 0.042). Similarly, a comprehensive assessment of RV, LA, and RA parameters revealed no significant differences in absolute or indexed values (all *p* > 0.05). Among all CMR parameters analyzed, LVEF was the only one that differed significantly between patients with and without subsequent MACE. Sex-stratified CMR parameters are detailed in Supplementary Material S5, revealing pronounced sex-based differences in ventricular volumes and mass, with males exhibiting larger cardiac dimensions even after BSA indexing.

**Table 2:** LV, RV, LA and RA parameters of cardiac magnetic resonance in patients.

Parameters	All Patients (*n* = 146)	MACE− (*n* = 110)	MACE+ (*n* = 36)	*p* value
LV EDV (mL)	112.9 ± 37.8	112.8 ± 36.8	113.6 ± 41.4	0.919
iLV EDV (mL/m^2^)	71.0 ± 15.9	71.2 ± 15.5	70.2 ± 17.4	0.723
LV ESV (mL)	44.2 ± 16.4	44.2 ± 17.2	44.5 ± 13.7	0.904
iLV ESV (mL/m^2^)	27.7 ± 7.1	27.5 ± 7.3	28.2 ± 6.8	0.608
LV SV (mL)	68.8 ± 23.7	69.4 ± 23.2	66.6 ± 25.4	0.537
iLV SV (mL/m^2^)	43.3 ± 10.7	43.8 ± 10.1	42.0 ± 12.7	0.386
LVEF (%)	60.9 ± 5.4	61.4 ± 5.2	59.3 ± 5.8	0.042
LV mass (g)	69.3 ± 27.8	69.6 ± 27.8	68.4 ± 28.1	0.812
iLV mass (g/m^2^)	42.7 ± 11.4	42.9 ± 11.1	42.4 ± 12.5	0.850
RV EDV (mL)	118.2 ± 41.4	119.5 ± 41.9	114.2 ± 40.0	0.508
iRV EDV (mL/m^2^)	74.1 ± 18.2	74.8 ± 16.5	72.2 ± 22.6	0.472
RV ESV (mL)	58.1 ± 23.7	58.1 ± 22.6	58.3 ± 27.3	0.959
iRV ESV (mL/m^2^)	36.6 ± 12.3	36.4 ± 10.0	37.1 ± 17.8	0.764
RV SV (mL)	60.0 ± 23.3	61.4 ± 24.0	55.9 ± 20.6	0.218
iRV SV (mL/m^2^)	37.5 ± 10.7	38.3 ± 10.7	35.1 ± 10.5	0.116
RVEF (%)	50.8 ± 8.7	51.3 ± 8.3	49.5 ± 10.1	0.287
iLA volume (mL/m^2^)	30.1 ± 10.2	29.3 ± 9.2	32.4 ± 12.8	0.115
iRA area (cm^2^/m^2^)	28.0 ± 9.0	27.5 ± 8.2	29.5 ± 11.2	0.262

*i* = indexed to BSA; BSA, body surface area; LV, left ventricular; EDV, end diastolic volume; ESV, end systolic volume; EF, ejection fraction; SV, stroke volume; RV, right ventricular; LA, left atrial; RA, right atrial.

### 3.2 Myocardial strain parameters

A comprehensive analysis of myocardial strain parameters was performed, comparing patients with and without MACE (Table 3 and Supplementary Material S6). Significant differences in global myocardial deformation patterns were observed between the MACE+ and MACE− groups. LV global radial strain (GRS) was significantly reduced in the MACE+ group (*p* = 0.004). Similarly, RV global circumferential strain (GCS) showed more pronounced impairment in MACE+ patients (*p* = 0.005). No significant differences were detected in LV GCS, LV global longitudinal strain (GLS), RV GRS, or RV GLS (all *p* > 0.05).

**Table 3:** Function parameters in patients recovering from myocarditis.

Parameters	All patients (*n* = 146)	MACE− (*n* = 110)	MACE+ (*n* = 36)	*p* Value
LV GRS (%)	39.1 ± 14.1	41.4 ± 14.3	33.9 ± 10.7	0.004
LV GCS (%)	−19.5 ± 3.7	−19.6 ± 3.9	−19.0 ± 3.2	0.424
LV GLS (%)	−15.5 ± 4.7	−15.5 ± 5.2	−15.5 ± 2.9	0.985
RV GRS (%)	52.5 ± 98.1	57.8 ± 111.6	36.4 ± 28.1	0.259
RV GCS (%)	−5.4 ± 19.1	−4.1 ± 21.5	−11.0 ± 7.7	0.005
RV GLS (%)	−11.6 ± 14.2	−11.1 ± 15.9	−13.2 ± 6.5	0.435
LV middle RS (%)	34.3 ± 13.9	36.1 ± 14.4	29.2 ± 7.7	0.006
RV apical CS (%)	−6.4 ± 25.8	−4.2 ± 28.6	−13.1 ± 11.9	0.010
LV systolic GRS rate (1/s)	2.4 ± 2.6	2.4 ± 3.0	2.4 ± 1.4	0.971
LV systolic GCS rate (1/s)	−1.1 ± 0.2	−1.1 ± 0.2	−1.1 ± 0.2	0.056
LV systolic GLS rate (1/s)	−0.9 ± 0.8	−1.0 ± 0.9	−0.7 ± 0.6	0.085
LV diastolic GRS rate (1/s)	−2.2 ± 2.7	−2.4 ± 2.8	−1.7 ± 2.4	0.256
LV diastolic GCS rate (1/s)	1.1 ± 0.5	1.1 ± 0.4	0.9 ± 0.6	0.003
LV diastolic GLS rate (1/s)	0.9 ± 0.7	0.9 ± 0.7	0.7 ± 0.7	0.153
RV systolic GRS rate (1/s)	2.7 ± 11.8	3.3 ± 13.6	1.2 ± 3.7	0.403
RV systolic GCS rate (1/s)	−0.8 ± 3.0	−0.9 ± 3.5	−0.6 ± 0.8	0.647
RV systolic GLS rate (1/s)	−0.8 ± 2.4	−0.9 ± 2.7	−0.7 ± 0.8	0.714
RV diastolic GRS rate (1/s)	0.9 ± 13.8	1.5 ± 15.7	−0.5 ± 5.8	0.486
RV diastolic GCS rate (1/s)	0.2 ± 2.5	0.3 ± 2.9	0.02 ± 1.2	0.562
RV diastolic GLS rate (1/s)	0.9 ± 2.6	1.0 ± 3.0	0.7 ± 0.6	0.670
LV diastolic middle CS rate (1/s)	1.1 ± 0.5	1.2 ± 0.4	0.9 ± 0.6	<0.001
LV diastolic apical CS rate (1/s)	1.4 ± 0.7	1.5 ± 0.6	1.1 ± 0.7	0.007
RV diastolic basal RS rate (1/s)	−0.4 ± 11.7	−1.5 ± 10.3	2.9 ± 14.8	0.045

LV, left ventricular; GRS, global radial strain; GCS, global circumferential strain; GLS, global longitudinal strain; RV, right ventricular; RS, radial strain; CS, circumferential strain; *p*1, compared female with male; *p*2, compare MACE− with MACE+.

Regional strain analysis revealed specific patterns of myocardial dysfunction. LV middle radial strain (RS) was significantly impaired in MACE+ patients compared to MACE− patients (*p* = 0.006). Additionally, RV apical circumferential strain (CS) demonstrated more substantial impairment in the MACE+ group relative to the MACE− group (*p* = 0.010). Other regional parameters, including basal and apical LV RS, did not show statistically significant differences between groups.

Analysis of myocardial deformation rates identified several significant differences. The LV diastolic CS rate was consistently reduced in MACE+ patients across multiple parameters: global LV diastolic GCS rate (*p* = 0.003), LV diastolic middle CS rate (*p* < 0.001), and LV diastolic apical CS rate (*p* = 0.007). The RV diastolic basal RS rate was significantly higher in MACE+ patients compared to MACE− patients (*p* = 0.045).

In summary, patients who experienced MACE following myocarditis exhibited significant impairments in both global and regional myocardial deformation, particularly affecting radial and circumferential strain components. The most pronounced differences were observed in LV GRS, RV GCS, and several diastolic strain rate parameters, suggesting that these measures hold prognostic value during recovery from myocarditis.

### 3.3 Correlation analyses among clinical, echocardiography variables and CMR parameters

**Figure 4:**
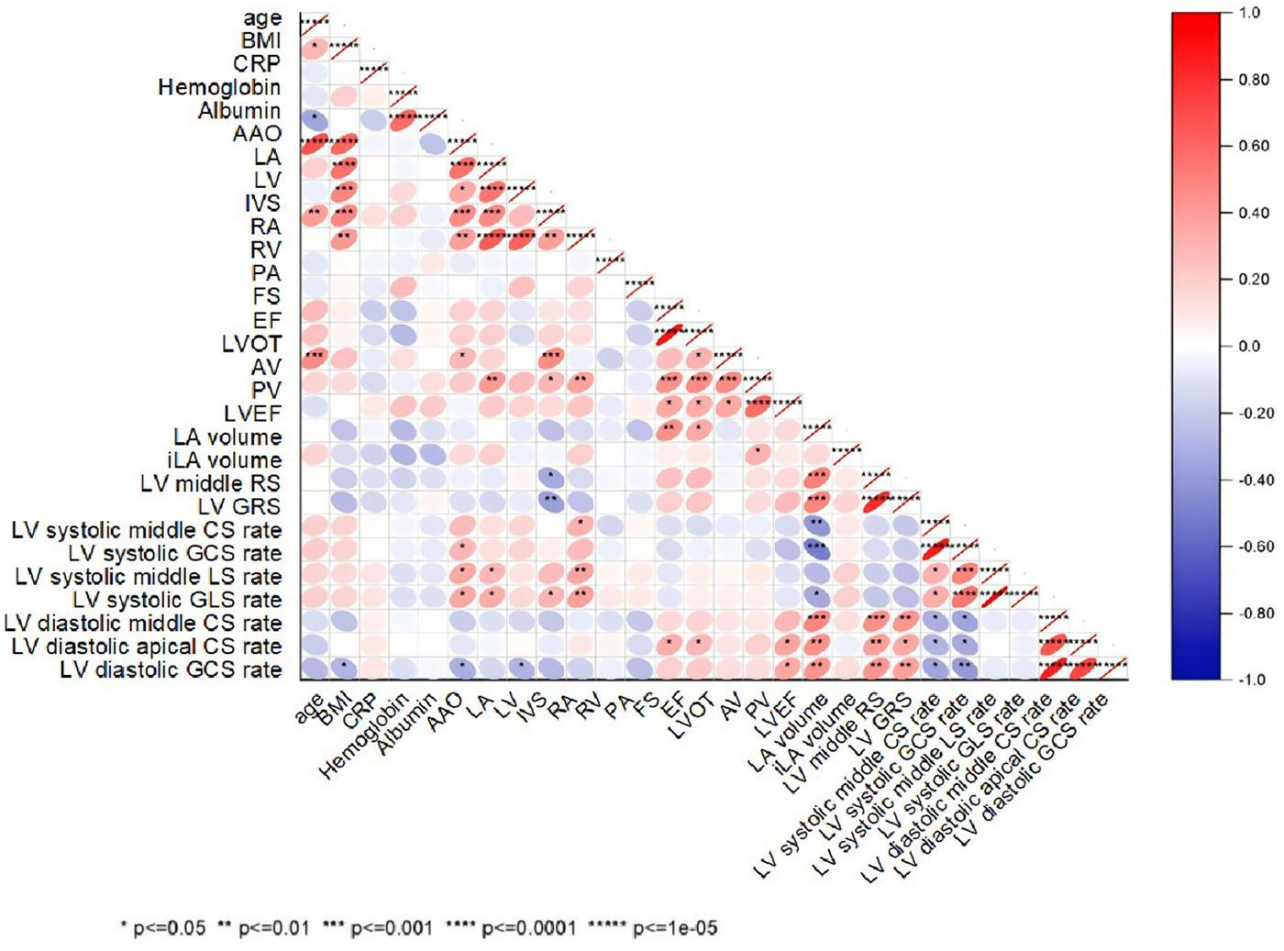
Correlation analyses among clinical, echocardiography variables and CMR parameters. BMI, body mass index; CRP, C-reactive protein; AAO, ascending aorta; LA, left atrial; LV, left ventricle; IVS, interventricular septum; RA, right atrium; RV, right ventricle; PA, pulmonary artery; FS, fraction shorting; EF, ejection fractions; LVOT, left ventricular outflow tract; AV, aortic valve; PV, pulmonary valve; RS, radial strain; GRS, global radial strain; CS, circumferential strain; GCS, global circumferential strain; LS, longitudinal strain; GLS, global longitudinal strain; *i* = indexed to body surface area.

LV middle RS (*r* = −0.325, *p* < 0.001) and LV GRS (*r* = −0.396, *p* < 0.001) showed modest but significant negative correlations with BMI. Modest correlations were also observed between echocardiographic measures (ascending aorta, LA, LV, interventricular septum, RA) and CMR parameters (LA volume, indexed LA volume, LV RS, and LV circumferential/longitudinal strain rates; *p* < 0.05), as illustrated in Figure 4.

### 3.4 LGE results

**Figure 5:**
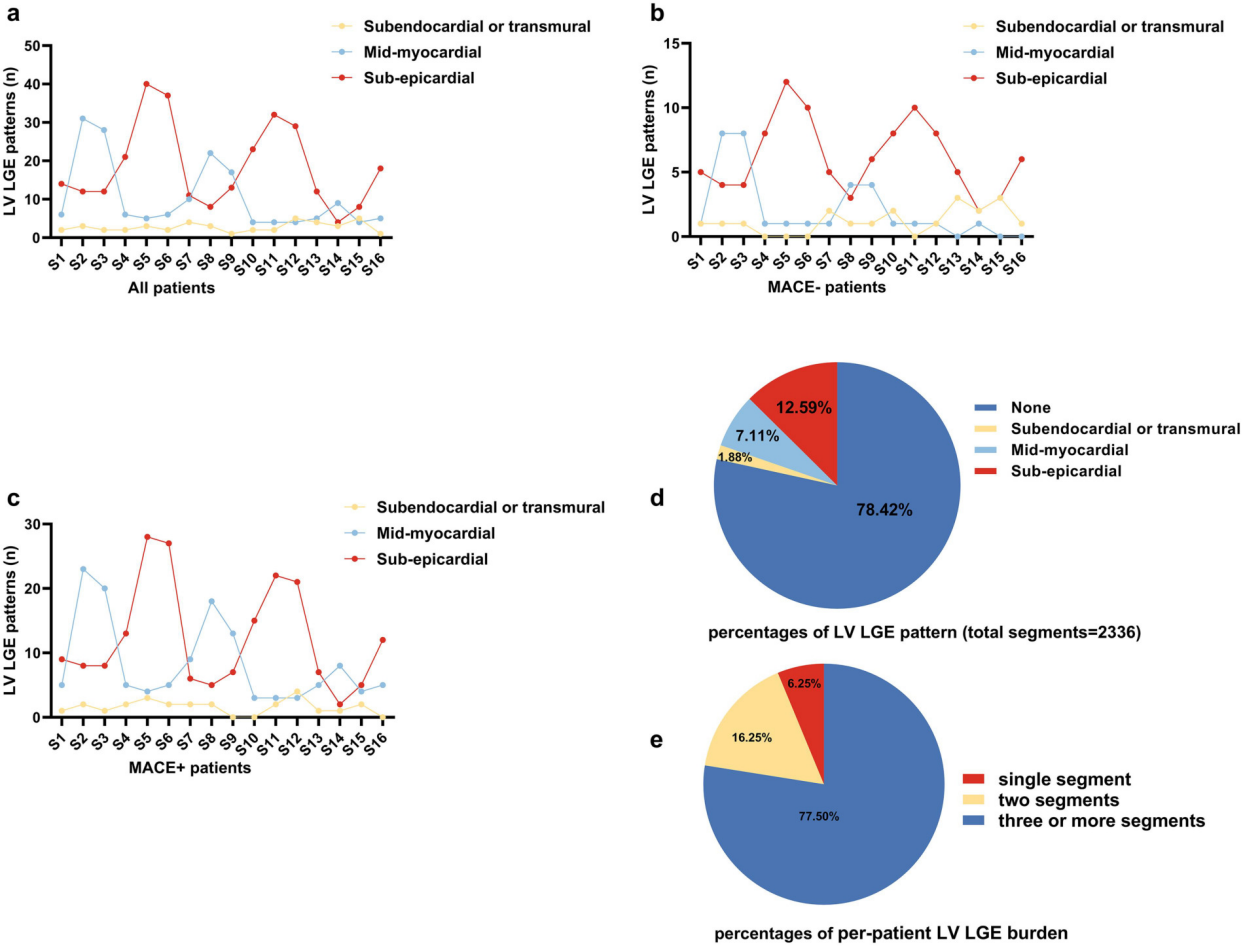
Left ventricular LGE characteristics in patients recovering from myocarditis. **(a)** Left ventricular LGE characteristics in all patients. **(b)** Left ventricular LGE characteristics in MACE− group. **(c)** Left ventricular LGE characteristics in MACE+ group. **(d)** Percentage of left ventricular LGE pattern. **(e)** Percentage of per-patient left ventricular LGE burden. LV Left ventricular; CMR, cardiovascular MRI; LGE, late gadolinium enhancement; MACE, major adverse cardiovascular event; Segments: 1: basal anterior, 2: basal anteroseptal, 3: basal inferoseptal, 4: basal inferior, 5: basal inferolateral, 6: basal anterolateral, 7: mid anterior, 8: mid anteroseptal, 9: mid inferoseptal, 10: mid inferior, 11: mid inferolateral, 12: mid anterolateral, 13: apical anterior, 14: apical septal, 15: apical inferior, 16: apical lateral.

Pericardial effusion was detected in 44 patients (30.1%), of which 41 were small and 3 were moderate. LGE predominantly affected the basal anteroseptal (S2, 31.5%), basal inferoseptal (S3, 28.8%), basal inferolateral (S5, 32.9%), basal anterolateral (S6, 30.8%), mid anteroseptal (S8, 22.6%), mid inferoseptal (S9, 21.2%), mid inferolateral (S11, 26.0%), and mid anterolateral (S12, 26.0%) segments. As shown in Figure 5a, sub-epicardial LGE was most frequently observed in segments S5 and S6, whereas mid-myocardial LGE was primarily involved in S2 and S3. These LGE characteristics were consistent across the two subgroups, as shown in Figures 5b,c. Figure 5d shows that the percentage of LGE was 12.59% in the sub-epicardial layer, 7.11% in the mid-myocardial layer, and only 1.88% in the subendocardial or transmural layer (total segments = 2,336). Furthermore, analysis of per-patient LGE burden revealed that among the 80 patients (54.80%) with detectable LGE, 5 (6.25% of LGE-positive patients) had single-segment involvement, 13 (16.25%) had two segments involved, and 62 (77.50%) had three or more segments involved (Figure 5e).

### 3.5 Clinical outcomes

**Figure 6:**
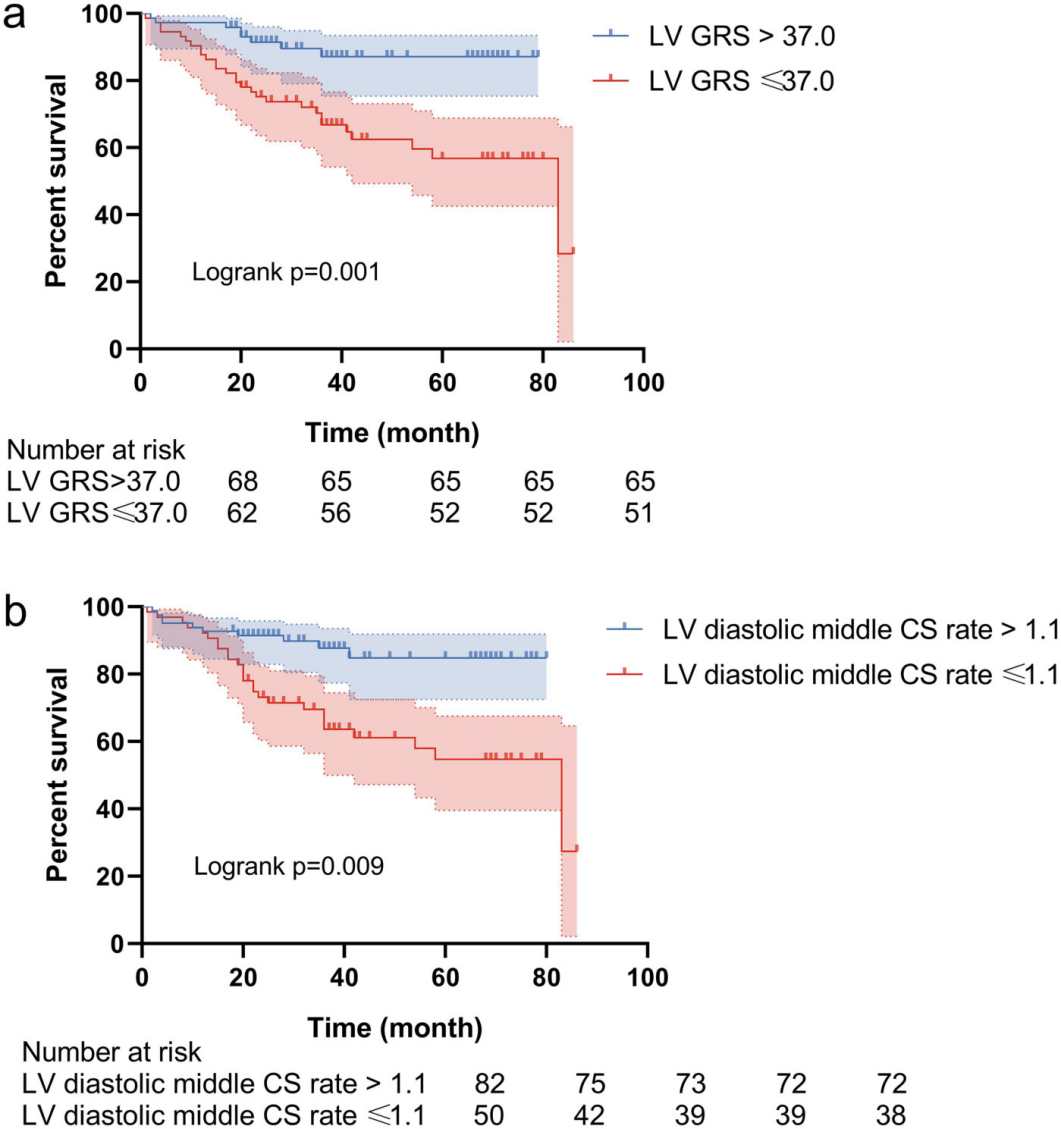
Kaplan–meier curve for the outcomes in patients recovering from myocarditis. LV Left ventricular; GRS, global radial strain; CS, circumferential strain.

Thirty-six patients (24.7%) experienced MACE, including 2 cases of cardiovascular death, 23 rehospitalizations, 6 heart failure events, and 5 cardiac surgeries. The median follow-up duration was 83.0 ± 17.7 months. ROC analysis identified optimal cutoff values for LV GRS (37%) and LV diastolic middle CS rate (1.1 1/s) based on the Youden index, effectively stratifying patients with and without subsequent MACE (Figure 6).

In univariate analysis, several parameters were significantly associated with MACE, including LV GRS [hazard ratio (HR): 0.961, 95% confidence interval (CI): 0.929–0.994; *p* = 0.019], LV middle CS (HR: 0.961, 95% CI: 0.927–0.996; *p* = 0.030), LV diastolic GCS rate (HR: 0.574, 95% CI: 0.351–0.939; *p* = 0.027), LV diastolic middle CS rate (HR: 0.561, 95% CI: 0.360–0.873; *p* = 0.010), and LV diastolic apical CS rate (HR: 0.676, 95% CI: 0.480 = 0.952; *p* = 0.025). Variables with *p* < 0.05 in univariate analysis (including LV GRS, LV middle RS, LV diastolic middle/apical CS rates and LGE presence were included in the multivariable Cox proportional hazards model (Table 4). No variables were excluded due to collinearity (all variance inflation factors <5.0). After adjustment, LV GRS (HR: 0.966, 95% CI: 0.937–0.996; *p* = 0.026) and LV diastolic middle CS rate (HR: 0.528, 95% CI: 0.317–0.879; *p* = 0.014) remained independent predictors of MACE. The results of univariable and multivariable Cox regression analyses are summarized in Table 4.

**Table 4:** Univariate and multivariate Cox regression analysis for prediction of MACE.

Outcome	Parameters	VIF	SE	Wald	HR (95% CI)	*p* value
Endpoints	Univariable	–	–	–	–	–
	LGE	1.037	0.365	2.574	1.795 (0.878–3.667)	0.109
	LV GRS	3.568	0.017	5.461	0.961 (0.929–0.994)	0.019
	LV middle RS	3.535	0.018	4.719	0.961 (0.927–0.996)	0.030
	LV diastolic middle CS rate	3.130	0.226	6.549	0.561 (0.360–0.873)	0.010
	LV diastolic apical CS rate	3.112	0.175	5.018	0.676 (0.480–0.952)	0.025
	Multivariable	–	–	–	–	–
	LV GRS	1	0.016	4.974	0.966 (0.937–0.996)	0.026
	LV diastolic middle CS rate	1	0.260	6.035	0.528 (0.317–0.879)	0.014
Hard endpoints	Univariable	–	–	–	–	–
	LGE	1.028	0.586	0.058	1.151 (0.365–3.633)	0.810
	LV GRS	3.574	0.034	7.987	0.910 (0.852–0.971)	0.005
	LV middle RS	3.537	0.035	5.351	0.922 (0.861–0.988)	0.021
	LV diastolic middle CS rate	1.020	0.215	10.546	0.498 (0.326–0.758)	0.001
	Multivariable	–	–	–	–	–
	LV GRS	1	0.035	4.776	0.927 (0.866–0.992)	0.029
	LV diastolic middle CS rate	1	0.269	7.836	0.471 (0.278–0.798)	0.005

HR, hazard ratio; CI, confidence interval; LGE, late gadolinium enhancement; LV, left ventricular; GRS, global radial strain; RS, radial strain; LS, longitudinal strain; CS, circumferential strain; MACE, major adverse cardiac events; VIF, variance inflation factor.

A sensitivity analysis using a stricter composite of hard endpoints (cardiovascular death, sustained ventricular arrhythmia, heart transplantation, or decline in LVEF to <50%) was performed. Among the 13 patients who experienced these endpoints, both LV GRS (HR: 0.927, 95% CI: 0.866–0.992, *p* = 0.029) and LV diastolic middle CS rate (HR: 0.471, 95% CI: 0.278–0.798, *p* = 0.005) remained independent predictors (Table 4). Additional subgroup analyses stratified by LGE status confirmed the robust prognostic performance of LV global RS (HR: 0.930, 95% CI: 0.886–0.977, *p* = 0.004) and RV middle longitudinal strain (HR: 0.909, 95% CI: 0.841–0.982, *p* = 0.015; Supplementary Material S7). These findings collectively demonstrate the robustness of our primary results.

## 4 Discussion

This study specifically investigated the in-hospital convalescent phase of myocarditis in patients with pLVEF. Our findings demonstrate that: (1) LV GRS and LV diastolic middle CS rate are independent prognostic factors in this patient population; and (2) LV strain parameters exhibit significant correlations with clinical and echocardiographic variables. These results underscore the potential clinical utility of LV GRS in the functional assessment of myocardial performance and highlight its value for risk stratification during the in-hospital convalescent phase.

Cardiac strain analysis is a reproducible, non-invasive technique with established prognostic value in myocarditis (14). Consistent with previous reports (10, 15, 16), we observed significantly reduced LV RS among patients who experienced MACE during follow-up. These alterations may reflect persistent subclinical myocardial dysfunction resulting from inflammatory changes, particularly within the mid-myocardial and sub-epicardial layers-regions critically involved in radial deformation (17). Impairment in these areas may compromise radial contractility, thereby providing a mechanistic explanation for the observed strain reduction (18).

In our cohort, conventional transthoracic echocardiography (TTE) parameters were largely within normal limits in most patients, consistent with the preserved systolic function required for inclusion. This finding underscores the limited sensitivity of routine echocardiography in detecting subtle residual myocardial dysfunction during the convalescent phase of myocarditis. In contrast, CMR-based strain analysis identified significant impairments in LV GRS and diastolic CS rate in those who experienced MACE, highlighting its incremental value in risk stratification beyond standard echocardiographic assessment. Thus, while TTE remains a valuable first-line tool for initial evaluation and follow-up, myocardial strain analysis provides a more sensitive means of identifying subclinical dysfunction and predicting adverse outcomes in this population.

Furthermore, our study reinforces the utility of CMR-derived strain parameters alongside LGE imaging in the evaluation of convalescent myocarditis patients with pLVEF. This is clinically pertinent, as residual myocardial inflammation may persist even after clinical recovery (15, 19). To our knowledge, the study by Brendel et al. (5) is among the few that has detailed both LGE and global strain characteristics in a cohort of 103 recovering myocarditis patients. The more favorable strain values observed in our cohort may reflect differences in inclusion criteria and patient selection. In addition to global strain assessment, we performed segmental analysis (basal, mid, apical), thereby extending previous observations and providing more granular characterization of myocardial involvement (20, 21). Strain rate represents the temporal derivative of strain, reflecting the velocity of myocardial deformation (22). Strain rate analysis can detect subtle functional alterations before observable changes in strain emerge, suggesting that changes in strain rate generally precede those in strain itself (23, 24). This may explain why the LV middle diastolic CS rate, but not the corresponding strain value, served as an independent predictor of outcomes in our study.

The prognostic implications of myocardial strain must also be interpreted in the context of our cohort's age profile. Established CMR feature-tracking reference values indicate that healthy young adults typically exhibit LV GRS values around 38.7%, LV GCS values approximately −21%, and LV GLS values of nearly −18.6% (25). The strain values in our overall cohort (LV GRS: 39.1%; LV GCS: −19.5%) fall within expected ranges for a young population. Importantly, however, the MACE+ subgroup showed significantly reduced LV GRS (33.7%) compared to both the MACE− subgroup and typical age-matched norms. This indicates that strain impairment retains prognostic relevance even in younger cohorts, underscoring its sensitivity in detecting clinically significant myocardial dysfunction independent of age-related changes (26). Future studies involving older myocarditis cohorts are warranted to explore potential age-related interactions in strain-based prognostication.

LGE is a well-established marker of myocardial injury and fibrosis. In our cohort, LGE was most frequently observed in the basal inferolateral segment, with enhancement patterns including sub-epicardial, mid-myocardial, and less commonly, subendocardial involvement. These findings are consistent with previous reports indicating that myocarditis can manifest ischemic-like injury patterns (27, 28). Although LGE extent was greater in the MACE+ group-potentially indicative of persistent inflammation or early fibrosis-it was not an independent predictor of MACE in our multivariate model. This may be attributable to the relatively young age and low burden of cardiovascular comorbidities in our cohort (29–32), where the overall myocardial reserve might be sufficient to compensate for focal injury, rendering functional parameters like strain more sensitive markers of impending risk. This aligns with emerging evidence that strain impairment often precedes or occurs independently of focal fibrosis in inflammatory cardiomyopathies, particularly in younger individuals with pLVEF. In such a population, the functional consequences of myocardial injury, as captured by strain impairment, may offer superior prognostic discrimination compared to the anatomical evidence of injury provided by LGE alone. Nevertheless, LGE remains a cornerstone in the diagnostic workup of suspected myocarditis due to its high specificity for tissue characterization.

Several factors may underlie the observed trends in sex-based outcome differences. These include variations in immune response and inflammatory regulation (33), which may lead to differences in myocardial remodeling and fibrosis despite similar conventional imaging markers (34). Delayed diagnosis or referral bias may also play a role, as women often present with more atypical symptoms, potentially leading to later intervention (35). Additionally, microvascular dysfunction or endothelial inflammation (36) may disproportionately affect women and contribute to adverse outcomes even in the presence of pLVEF.

## 5 Limitation

Several limitations of our study must be acknowledged. First, the retrospective, single-center design and limited sample size may affect the generalizability of the findings. Second, endomyocardial biopsy, the diagnostic gold standard, was not performed in all cases, precluding histological confirmation and etiological subtyping. Future studies should incorporate molecular profiling to elucidate etiology-dependent outcomes. Third, 54 patients (27% of the eligible cohort) were lost to follow-up, primarily due to migration or loss of contact. Although the comparison of baseline characteristics between the analyzed cohort and those lost to follow-up revealed no significant differences (Supplementary Material S3), suggesting that missingness may be random for the measured covariates, we cannot rule out the possibility of unmeasured confounding factors related to the loss. The relatively low rate of hard endpoints further necessitates cautious interpretation, and future prospective studies should prioritize strategies to minimize attrition. Finally, our study population was predominantly young with a low burden of cardiovascular risk factors, which may limit the extrapolation of our results to older or more comorbid populations.

## 6 Conclusion

In conclusion, our findings support the incremental prognostic value of CMR-based myocardial strain analysis for risk stratification in patients recovering from myocarditis with pLVEF. LV GRS and LV diastolic middle CS rate emerged as independent predictors of MACE, highlighting their role in identifying residual myocardial dysfunction beyond conventional volumetric and functional measures.

## Data Availability

The original contributions presented in the study are included in the article/Supplementary Material, further inquiries can be directed to the corresponding author/s.
