# Supplementary material for: Myocarditis with preserved left ventricular ejection fraction in-hospital convalescent phase: insights from CMR and myocardial strain analysis
Source: Front Cardiovasc Med. 2026 Jan 12;12:1613394. doi: 10.3389/fcvm.2025.1613394 (PMC12833385; doi:10.3389/fcvm.2025.1613394)
Supplement: Supplementary file 1 [file Datasheet1.docx]

Supplementary Material

# Supplementary Material S1

**Table 1: Detailed scanning parameters of MRI.**

| **Parameters** | **1.5 T** | |  | **3.0 T** | |
| --- | --- | --- | --- | --- | --- |
|  | **Cine images** | **LGE images** |  | **Cine images** | **LGE images** |
| Section thickness | 5-8 mm | 8 mm |  | 5-8 mm | 8 mm |
| Gap | 1.2 | 1.6 mm |  | 2 mm | 2 mm |
| Repetition time | 49.8 msec | 870 msec |  | 34 msec | 589 msec |
| Echo time | 1.29 msec | 1.36 msec |  | 1.51 msec | 1.19 msec |
| Flip angle | 80 | 80 |  | 59 | 55 |
| Field of view | 340×330 mm^2^ | 360×270 mm^2^ |  | 340×380 mm^2^ | 360×270 mm^2^ |
| Temporal resolution | 18–32 msec | 679 msec |  | 20-35 msec | 627 msec |
| Acceleration factor | 2 | 2 |  | 3 | 3 |

Note: T, Tesla; LGE, late gadolinium enhancement.

# Supplementary Material S2

**Table 2: Intra- and inter-observer reproducibility of strain parameters.**

| Parameters | Intraobserver | | Interobserver | |
| --- | --- | --- | --- | --- |
|  | ICC | 95% CI | ICC | 95% CI |
| LV EDV (ml) | 0.99 | 0.98-0.99 | 0.97 | 0.93-0.98 |
| LV ESV (ml) | 0.93 | 0.76-0.96 | 0.93 | 0.82-0.90 |
| LV SV (ml) | 0.98 | 0.96-0.99 | 0.92 | 0.80-0.96 |
| LVEF (%) | 0.95 | 0.88-0.98 | 0.98 | 0.97-0.99 |
| LV mass (g) | 0.96 | 0.90-0.98 | 0.86 | 0.66-0.94 |
| RV EDV (ml) | 0.90 | 0.78-0.96 | 0.94 | 0.86-0.97 |
| RV ESV (ml) | 0.90 | 0.77-0.96 | 0.92 | 0.80-0.96 |
| RV SV (ml) | 0.89 | 0.75-0.95 | 0.85 | 0.63-0.94 |
| RVEF (%) | 0.89 | 0.75-0.96 | 0.89 | 0.75-0.96 |
| LA volume (ml) | 0.86 | 0.64-0.95 | 0.96 | 0.91-0.98 |
| LV basal RS (%) | 0.88 | 0.72-0.95 | 0.93 | 0.83-0.97 |
| LV middle RS (%) | 0.91 | 0.78-0.96 | 0.85 | 0.61-0.94 |
| LV apical RS (%) | 0.97 | 0.92-0.98 | 0.95 | 0.89-0.98 |
| LV GRS (%) | 0.95 | 0.88-0.98 | 0.89 | 0.75-0.96 |
| LV basal CS (%) | 0.86 | 0.68-0.94 | 0.81 | 0.59-0.92 |
| LV middle CS (%) | 0.99 | 0.97-0.99 | 0.93 | 0.83-0.97 |
| LV apical CS (%) | 0.96 | 0.90-0.98 | 0.90 | 0.77-0.96 |
| LV GCS (%) | 0.93 | 0.84-0.97 | 0.91 | 0.78-0.96 |
| LV basal LS (%) | 0.82 | 0.53-0.93 | 0.80 | 0.52-0.92 |
| LV middle LS (%) | 0.88 | 0.73-0.94 | 0.85 | 0.66-0.93 |
| LV apical LS (%) | 0.82 | 0.68-0.91 | 0.81 | 0.63-0.90 |
| LV GLS (%) | 0.88 | 0.74-0.95 | 0.85 | 0.67-0.92 |
| RV basal RS (%) | 0.97 | 0.94-0.99 | 0.97 | 0.93-0.99 |
| RV middle RS (%) | 0.98 | 0.97-0.99 | 0.98 | 0.96-0.99 |
| RV apical RS (%) | 0.83 | 0.57-0.93 | 0.83 | 0.57-0.93 |
| RV GRS (%) | 0.91 | 0.78-0.95 | 0.90 | 0.77-0.96 |
| RV basal CS (%) | 0.83 | 0.56-0.93 | 0.77 | 0.51-0.90 |
| RV middle CS (%) | 0.97 | 0.94-0.99 | 0.96 | 0.89-0.98 |
| RV apical CS (%) | 0.90 | 0.77-0.95 | 0.86 | 0.67-0.93 |
| RV GCS (%) | 0.91 | 0.79-0.96 | 0.88 | 0.73-0.94 |
| RV basal LS (%) | 0.96 | 0.91-0.98 | 0.91 | 0.80-0.96 |
| RV middle LS (%) | 0.98 | 0.95-0.99 | 0.95 | 0.89-0.98 |
| RV apical LS (%) | 0.94 | 0.89-0.97 | 0.83 | 0.61-0.93 |
| RV GLS (%) | 0.93 | 0.81-0.96 | 0.91 | 0.76-0.96 |
| LAS (%) | 0.93 | 0.83-0.97 | 0.88 | 0.72-0.95 |
| RAS (%) | 0.91 | 0.79-0.96 | 0.87 | 0.70-0.95 |
| LV systolic basal RS rate (1/s) | 0.89 | 0.71-0.96 | 0.81 | 0.55-0.93 |
| LV systolic middle RS rate (1/s) | 0.89 | 0.69-0.96 | 0.80 | 0.52-0.92 |
| LV systolic apical RS rate (1/s) | 0.86 | 0.61-0.95 | 0.75 | 0.45-0.90 |
| LV systolic GRS rate (1/s) | 0.94 | 0.85-0.98 | 0.90 | 0.75-0.96 |
| LV systolic basal CS rate (1/s) | 0.97 | 0.93-0.99 | 0.95 | 0.88-0.98 |
| LV systolic middle CS rate (1/s) | 0.94 | 0.85-0.98 | 0.89 | 0.74-0.96 |
| LV systolic apical CS rate (1/s) | 0.91 | 0.79-0.96 | 0.87 | 0.71-0.95 |
| LV systolic GCS rate (1/s) | 0.90 | 0.74-0.96 | 0.82 | 0.68-0.93 |
| LV systolic basal LS rate (1/s) | 0.88 | 0.72-0.94 | 0.83 | 0.70-0.94 |
| LV systolic middle LS rate (1/s) | 0.87 | 0.70-0.93 | 0.81 | 0.70-0.92 |
| LV systolic apical LS rate (1/s) | 0.87 | 0.71-0.93 | 0.83 | 0.71-0.93 |
| LV systolic GLS rate (1/s) | 0.81 | 0.70-0.93 | 0.79 | 0.55-0.92 |
| LV diastolic basal RS rate (1/s) | 0.79 | 0.53-0.90 | 0.77 | 0.52-0.91 |
| LV diastolic middle RS rate (1/s) | 0.77 | 0.51-0.90 | 0.77 | 0.51-0.90 |
| LV diastolic apical RS rate (1/s) | 0.80 | 0.68-0.91 | 0.81 | 0.70-0.92 |
| LV diastolic GRS rate (1/s) | 0.81 | 0.70-0.93 | 0.79 | 0.55-0.92 |
| LV diastolic basal CS rate (1/s) | 0.82 | 0.53-0.93 | 0.80 | 0.52-0.92 |
| LV diastolic middle CS rate (1/s) | 0.88 | 0.73-0.94 | 0.85 | 0.66-0.93 |
| LV diastolic apical CS rate (1/s) | 0.82 | 0.53-0.93 | 0.82 | 0.53-0.93 |
| LV diastolic GCS rate (1/s) | 0.88 | 0.73-0.94 | 0.83 | 0.56-0.93 |
| LV diastolic basal LS rate (1/s) | 0.91 | 0.79-0.96 | 0.87 | 0.71-0.95 |
| LV diastolic MLS rate (1/s) | 0.94 | 0.85-0.98 | 0.90 | 0.75-0.96 |
| LV diastolic ALS rate (1/s) | 0.94 | 0.89-0.97 | 0.83 | 0.61-0.93 |
| LV diastolic GLS rate (1/s) | 0.93 | 0.81-0.96 | 0.91 | 0.76-0.96 |
| RV systolic basal RS rate (1/s) | 0.93 | 0.83-0.97 | 0.89 | 0.69-0.96 |
| RV systolic middle RS rate (1/s) | 0.88 | 0.72-0.95 | 0.80 | 0.52-0.92 |
| RV systolic apical RS rate (1/s) | 0.86 | 0.61-0.95 | 0.79 | 0.55-0.92 |
| RV systolic GRS rate (1/s) | 0.87 | 0.70-0.93 | 0.82 | 0.68-0.93 |
| RV systolic basal CS rate (1/s) | 0.93 | 0.83-0.97 | 0.83 | 0.61-0.93 |
| RV systolic middle CS rate (1/s) | 0.94 | 0.85-0.98 | 0.89 | 0.69-0.96 |
| RV systolic apical CS rate (1/s) | 0.91 | 0.79-0.96 | 0.87 | 0.71-0.95 |
| RV systolic GCS rate (1/s) | 0.91 | 0.79-0.96 | 0.83 | 0.61-0.93 |
| RV systolic basal LS rate (1/s) | 0.89 | 0.75-0.96 | 0.82 | 0.53-0.93 |
| RV systolic middle LS rate (1/s) | 0.83 | 0.56-0.93 | 0.79 | 0.55-0.92 |
| RV systolic apical LS rate (1/s) | 0.85 | 0.66-0.93 | 0.82 | 0.53-0.93 |
| RV systolic GLS rate (1/s) | 0.81 | 0.70-0.92 | 0.79 | 0.55-0.92 |
| RV diastolic basal RS rate (1/s) | 0.91 | 0.79-0.96 | 0.87 | 0.71-0.95 |
| RV diastolic middle RS rate (1/s) | 0.93 | 0.81-0.96 | 0.89 | 0.74-0.96 |
| RV diastolic apical RS rate (1/s) | 0.88 | 0.72-0.95 | 0.83 | 0.71-0.93 |
| RV diastolic GRS rate (1/s) | 0.91 | 0.79-0.96 | 0.82 | 0.68-0.93 |
| RV diastolic basal CS rate (1/s) | 0.81 | 0.70-0.92 | 0.79 | 0.53-0.90 |
| RV diastolic middle CS rate (1/s) | 0.77 | 0.52-0.91 | 0.77 | 0.51-0.90 |
| RV diastolic apical CS rate (1/s) | 0.80 | 0.68-0.91 | 0.78 | 0.52-0.90 |
| RV diastolic GCS rate (1/s) | 0.81 | 0.70-0.93 | 0.79 | 0.55-0.92 |
| RV diastolic basal LS rate (1/s) | 0.90 | 0.78-0.96 | 0.94 | 0.86-0.97 |
| RV diastolic middle LS rate (1/s) | 0.89 | 0.75-0.96 | 0.89 | 0.75-0.95 |
| RV diastolic apical LS rate (1/s) | 0.89 | 0.75-0.96 | 0.85 | 0.63-0.94 |
| RV diastolic GLS rate (1/s) | 0.85 | 0.63-0.93 | 0.83 | 0.70-0.94 |

Note: ICC, intra-and interclass coefficient; CI, confidence interval; LV, left ventricular; RV, right ventricular; EDV, end diastolic volume; ESV, end systolic volume; EF, ejection fraction; SV, stroke volume; LA, left atrial; RA, right atrial; RS, radial strain; CS, circumferential strain; LS, longitudinal strain; GRS, global radial strain; GCS, global circumferential strain; GLS, global longitudinal strain.

# Supplementary Material S3

**Table 3: Baseline characteristics between the included patients and lost to follow-up patients.**

| **Characteristics** | **Included (n=146)** | **Lost (n=54)** | ***p* Value** |
| --- | --- | --- | --- |
| **Demographics** | - | - | - |
| Age (year) | 26±16 | 29±18 | 0.257 |
| Gender (Male, %) | 71 (48.6) | 28 (51.9) | 0.687 |
| Body mass index | 21.2±4.0 | 21.5±3.8 | 0.525 |
| **Cardiovascular risk factors** | - | - | - |
| Smoking (Yes, %) | 12 (8.2) | 5 (9.3) | 0.185 |
| Drinking (Yes, %) | 5 (3.4) | 3 (5.6) | 0.495 |
| Hypertension (Yes, %) | 11 (7.5) | 4 (7.4) | 0.976 |
| Diabetes (Yes, %) | 1 (0.7) | 1 (1.9) | 0.462 |
| Dyslipidemia (Yes, %) | 2 (1.4) | 1 (1.9) | 0.803 |
| **Laboratory results** | - |  |  |
| SBP (mmHg) | 117±16 | 121±13 | 0.101 |
| DBP (mmHg) | 72±11 | 69±14 | 0.114 |
| Hematocrit (%) | 39.2±4.0 | 38.5±5.2 | 0.314 |
| WBC (10^9^/L) | 6.9±2.1 | 6.7±1.9 | 0.541 |
| GFR (ml/min) | 131.2±27.7 | 132.3±22.4 | 0.794 |
| CRP (mg/L) | 16.5±64.0 | 17.3±56.2 | 0.936 |
| Hemoglobin (g/L) | 130.4±14.6 | 133.5±13.6 | 0.176 |
| CK-MB (U/L) | 25.0±31.4 | 26.2±29.3 | 0.807 |
| Albumin (g/L) | 41.1±4.3 | 42.3±3.9 | 0.074 |
| **Medication use (n, %)** | - | - | - |
| β-blocker | 59 (40.4) | 21 (38.9) | 0.845 |
| ACE inhibitor or ARBs | 105 (71.9) | 32 (59.3) | 0.087 |
| Steroids | 48 (32.9) | 17 (31.5) | 0.852 |
| Intravenous immunoglobulin | 28 (19.2) | 9 (16.7) | 0.685 |
| Inotropes | 135 (92.5) | 48 (88.9) | 0.421 |
| **ECG (n, %)** | - | - | - |
| Sinus rhythm | 67 (45.9) | 24 (44.4) | 0.855 |
| Sinus tachycardia | 22 (15.1) | 8 (14.8) | 0.964 |
| Sinus bradycardia | 13 (8.9) | 4 (7.4) | 0.736 |
| ST-changes | 19 (13.0) | 8 (14.8) | 0.741 |
| Pathologic Q wave | 3 (2.1) | 2 (3.7) | 0.507 |
| Ventricular arrhythmia | 10 (6.8) | 4 (7.4) | 0.891 |
| Left bundle branch block | 3 (2.1) | 2 (3.7) | 0.507 |
| Atrial Premature | 8 (5.5) | 3 (5.6) | 0.983 |
| Atrioventricular block | 1 (0.7) | 1 (1.9) | 0.462 |
| **TTE** | - | - | - |
| Ejection fractions (%) | 64.0±6.0 | 65.1±5.8 | 0.916 |
| Regional wall motion abnormalities (n, %) | 4 (2.7) | 2 (3.7) | 0.723 |

Note: SBP, systolic blood pressure; DBP, diastolic blood pressure; BNP, brain natriuretic peptide; WBC, white blood cell; GFR, glomerular filtration rate; CRP, C-reactive protein; CK-MB, creatine kinase isoenzymes; ACE indicates angiotensin-converting enzyme; ARB, angiotensin II receptor blocker; ECG, electrocardiogram; TTE, transthoracic echocardiography.

# Supplementary Material S4

**Table 4:** **Echocardiography data of patients**

| **Parameters** | **All Patients (n=146)** | **MACE- (n=110)** | **MACE+ (n=36)** | ***P* Value** |
| --- | --- | --- | --- | --- |
| AAO (cm) | 2.61±0.50 | 2.60±0.48 | 2.63±0.55 | 0.754 |
| LA (cm) | 3.06±0.57 | 3.01±0.53 | 3.21±0.67 | 0.068 |
| LV (cm) | 4.42±0.58 | 4.40±0.56 | 4.47±0.63 | 0.529 |
| IVS (cm) | 0.83±0.18 | 0.83±0.18 | 0.82±0.19 | 0.776 |
| RA (cm) | 3.38±0.50 | 3.38±0.49 | 3.37±0.54 | 0.918 |
| RV (cm) | 3.28±0.51 | 3.29±0.49 | 3.26±0.57 | 0.760 |
| PA (cm) | 2.36±1.81 | 2.41±2.09 | 2.19±0.35 | 0.532 |
| FS (%) | 34.91±4.93 | 34.67±5.12 | 35.58±4.33 | 0.339 |
| EF (%) | 64.02±6.02 | 63.61±6.15 | 65.23±5.50 | 0.162 |
| E’/A’ or E/A (<1, %) | 10 (6.8) | 7 (6.4) | 3 (8.3) | 0.685 |
| LVOT (m/s) | 0.87±0.26 | 0.86±0.23 | 0.90±0.31 | 0.409 |
| AV (m/s) | 1.19±0.26 | 1.21±0.27 | 1.15±0.26 | 0.245 |
| PV (m/s) | 0.93±0.18 | 0.94±0.18 | 0.89±0.17 | 0.145 |
| Mitral regurgitation (Yes, %) | 43 (29.5) | 32+1 (30) | 10 (27.8) | 0.800 |
| Aortic regurgitation (Yes, %) | 13 (8.9) | 8 (7.3) | 4+1 (13.9) | 0.226 |
| Tricuspid regurgitation (Yes, %) | 75 (51.4) | 57 (51.8) | 15+3 (50.0) | 0.850 |
| Pulmonary regurgitation (Yes, %) | 7 (4.8) | 4 (3.6) | 3 (8.3) | 0.252 |
| Regional wall motion abnormalities | 4 (2.7) | 1 (0.9) | 3 (8.3) | 0.018 |
| Ventricular wall motion score | 1.00±0.19 | 1.00±0.15 | 1.01±0.27 | 0.780 |

Note: MACE, major adverse cardiac events; AAO, ascending aorta; LA, left atrial; LV, left ventricle; IVS, interventricular septum; RA, right atrium; RV, right ventricle; PA, pulmonary artery; FS, fraction shorting; EF, ejection fractions; LVOT, left ventricular outflow tract; AV, aortic valve; PV, pulmonary valve.

# Supplementary Material S5

**Table 5: LV, RV, LA and RA parameters of cardiac magnetic resonance in male and female patients.**

| **Parameters** | **All Patients (n=146)** | **Female (n=75)** | **Male (n=71)** | ***p* value** |
| --- | --- | --- | --- | --- |
| LV EDV (ml) | 112.9±37.8 | 106.0±37.1 | 120.4±37.4 | 0.021 |
| iLV EDV (ml/m^2^) | 71.0±15.9 | 66.6±15.5 | 75.7±15.2 | <0.001 |
| LV ESV (ml) | 44.2±16.4 | 38.4±11.4 | 50.4±18.6 | <0.001 |
| iLV ESV (ml/m^2^) | 27.7±7.1 | 26.0±6.5 | 29.5±7.3 | 0.003 |
| LV SV (ml) | 68.8±23.7 | 60.0±18.0 | 78.0±25.4 | <0.001 |
| iLV SV (ml/m^2^) | 43.3±10.7 | 40.6±10.7 | 46.2±10.1 | 0.001 |
| LVEF (%) | 60.9±5.4 | 60.8±5.2 | 61.1±5.6 | 0.721 |
| LV mass (g) | 69.3±27.8 | 57.4±20.1 | 81.9±29.3 | <0.001 |
| iLV mass (g/m^2^) | 42.7±11.4 | 38.1±9.3 | 47.8±11.4 | <0.001 |
| RV EDV (ml) | 118.2±41.4 | 101.8±31.9 | 135.5±43.4 | <0.001 |
| iRV EDV (ml/m^2^) | 74.1±18.2 | 68.8±19.1 | 79.8±15.2 | <0.001 |
| RV ESV (ml) | 58.1±23.7 | 49.8±20.3 | 67.0±24.0 | <0.001 |
| iRV ESV (ml/m^2^) | 36.6±12.3 | 33.9±13.9 | 39.5±9.8 | 0.006 |
| RV SV (ml) | 60.0±23.3 | 52.1±18.5 | 68.5±24.9 | <0.001 |
| iRV SV (ml/m^2^) | 37.5±10.7 | 34.9±10.4 | 40.3±10.4 | 0.002 |
| RVEF (%) | 50.8±8.7 | 50.1±9.2 | 50.5±8.3 | 0.697 |
| iLA volume (ml/m^2^) | 30.1±10.2 | 29.9±10.7 | 30.3±9.8 | 0.789 |
| iRA area (cm^2^/m^2^) | 28.0±9.0 | 27.0±8.7 | 29.0±9.3 | 0.203 |

Note: i = indexed to BSA; LV, left ventricular; EDV, end diastolic volume; ESV, end systolic volume; EF, ejection fraction; SV, stroke volume; RV, right ventricular; LA, left atrial; RA, right atrial; BSA, body surface area; p1, compared female with male; p2, compare MACE- with MACE+.

# Supplementary Material S6

**Table 6: Function parameters of strain in patients recovering from myocarditis.**

| **Parameters** | **All Patients (n=146)** | **MACE- (n=110)** | **MACE+ (n=36)** | ***p* value** |
| --- | --- | --- | --- | --- |
| LV basal RS (%) | 55.6±28.9 | 58.1±31.6 | 48.1±17.0 | 0.081 |
| LV middle RS (%) | 34.3±13.9 | 36.1±14.4 | 29.2±7.7 | 0.006 |
| LV apical RS (%) | 45.4±37.2 | 47.8±38.9 | 38.4±31.1 | 0.204 |
| LV basal CS (%) | -18.9±2.7 | -19.0±2.5 | -18.7±3.0 | 0.533 |
| LV middle CS (%) | -19.7±3.0 | -19.9±2.9 | -19.0±3.3 | 0.130 |
| LV apical CS (%) | -20.8±4.8 | -20.0±5.0 | -20.3±4.2 | 0.529 |
| LV basal LS (%) | -14.8±5.2 | -14.7±5.7 | -14.9±3.5 | 0.910 |
| LV middle LS (%) | -15.2±5.2 | -15.2±5.7 | -15.3±3.2 | 0.862 |
| LV apical LS (%) | -17.0±4.4 | -17.1±4.7 | -16.7±3.1 | 0.675 |
| RV basal RS (%) | 68.5±99.6 | 73.6±111.0 | 52.9±48.6 | 0.281 |
| RV middle RS (%) | 63.9±177.7 | 72.3±203.2 | 38.2±36.0 | 0.320 |
| RV apical RS (%) | 48.8±132.4 | 55.4±151.1 | 28.5±30.1 | 0.293 |
| RV basal CS (%) | -1.6±23.1 | -0.1±26.0 | -6.2±9.2 | 0.167 |
| RV middle CS (%) | -5.2±28.7 | -3.6±32.5 | -10.1±9.0 | 0.242 |
| RV apical CS (%) | -6.4±25.8 | -4.2±28.6 | -13.1±11.9 | 0.010 |
| RV basal LS (%) | -8.8±15.4 | -8.3±17.1 | -10.4±8.6 | 0.474 |
| RV middle LS (%) | -8.5±25.6 | -7.4±29.1 | -11.9±8.5 | 0.360 |
| RV apical LS (%) | -14.5±22.8 | -13.8±25.8 | -16.5±9.5 | 0.552 |
| LV systolic basal RS rate (1/s) | 2.8±5.1 | 2.8±5.9 | 2.7±1.3 | 0.923 |
| LV systolic middle RS rate (1/s) | 1.7±3.1 | 1.6±3.5 | 1.7±1.4 | 0.898 |
| LV systolic apical RS rate (1/s) | 2.4±7.0 | 1.8±7.8 | 3.8±4.4 | 0.193 |
| LV systolic basal CS rate (1/s) | -1.0±0.4 | -1.1±0.4 | -1.0±0.2 | 0.411 |
| LV systolic middle CS rate (1/s) | -1.1±0.3 | -1.1±0.3 | -1.0±0.2 | 0.022 |
| LV systolic apical CS rate (1/s) | -1.3±0.4 | -1.3±0.4 | -1.2±0.3 | 0.103 |
| LV systolic basal LS rate (1/s) | -0.9±1.0 | -1.0±1.1 | -0.7±0.6 | 0.185 |
| LV systolic middle LS rate (1/s) | -1.0±0.9 | -1.1±1.0 | -0.7±0.6 | 0.082 |
| LV systolic apical LS rate (1/s) | -1.0±0.7 | -1.0±0.7 | -0.9±0.4 | 0.386 |
| LV diastolic basal RS rate (1/s) | -4.2±4.4 | -3.8±4.7 | -5.1±3.6 | 0.180 |
| LV diastolic middle RS rate (1/s) | -1.9±2.7 | -1.9±3.0 | -1.9±1.5 | 0.976 |
| LV diastolic apical RS rate (1/s) | -2.2±6.5 | -2.3±7.1 | -2.0±4.9 | 0.865 |
| LV diastolic basal CS rate (1/s) | 1.0±0.6 | 1.0±0.7 | 1.0±0.5 | 0.916 |
| LV diastolic middle CS rate (1/s) | 1.1±0.5 | 1.2±0.4 | 0.9±0.6 | <0.001 |
| LV diastolic apical CS rate (1/s) | 1.4±0.7 | 1.5±0.6 | 1.1±0.7 | 0.007 |
| LV diastolic basal LS rate (1/s) | 0.8±0.9 | 0.8±0.9 | 0.8±0.8 | 0.800 |
| LV diastolic middle LS rate (1/s) | 0.9±1.0 | 0.9±1.0 | 0.8±0.9 | 0.682 |
| LV diastolic apical LS rate (1/s) | 1.1±0.5 | 1.1±0.5 | 0.9±0.4 | 0.110 |
| RV systolic basal RS rate (1/s) | 2.2±18.3 | 2.6±21.2 | 1.3±5.3 | 0.736 |
| RV systolic middle RS rate (1/s) | 2.2±10.6 | 2.5±11.9 | 1.3±5.4 | 0.593 |
| RV systolic apical RS rate (1/s) | 5.3±34.7 | 6.8±40.5 | 1.1±5.1 | 0.430 |
| RV systolic basal CS rate (1/s) | -1.0±4.9 | -1.2±5.6 | -0.4±1.2 | 0.390 |
| RV systolic middle CS rate (1/s) | -1.3±4.5 | -1.5±5.2 | -0.6±1.0 | 0.342 |
| RV systolic apical CS rate (1/s) | -0.5±5.3 | -0.3±6.1 | -0.8±1.2 | 0.706 |
| RV systolic basal LS rate (1/s) | -0.8±2.8 | -0.9±3.1 | -0.4±1.3 | 0.397 |
| RV systolic middle LS rate (1/s) | -1.1±4.6 | -1.2±5.3 | -0.8±0.9 | 0.604 |
| RV systolic apical LS rate (1/s) | -0.9±3.7 | -0.9±4.3 | -0.8±1.3 | 0.869 |
| RV diastolic basal RS rate (1/s) | -0.4±11.7 | -1.5±10.3 | 2.9±14.8 | 0.045 |
| RV diastolic middle RS rate (1/s) | 0.8±13.8 | 1.2±15.6 | -0.4±6.6 | 0.583 |
| RV diastolic apical RS rate (1/s) | 3.9±52.7 | 5.7±61.5 | -0.9±5.5 | 0.549 |
| RV diastolic basal CS rate (1/s) | 0.3±2.1 | 0.3±2.3 | 0.3±1.6 | 0.948 |
| RV diastolic middle CS rate (1/s) | 1.0±3.7 | 1.3±4.3 | 0.3±1.4 | 0.219 |
| RV diastolic apical CS rate (1/s) | 0.8±5.7 | 1.0±6.6 | 0.3±1.7 | 0.545 |
| RV diastolic basal LS rate (1/s) | 0.7±2.1 | 0.7±2.3 | 0.5±1.4 | 0.616 |
| RV diastolic middle LS rate (1/s) | 1.1±4.7 | 1.3±5.5 | 0.7±0.8 | 0.582 |
| RV diastolic apical LS rate (1/s) | 1.0±3.5 | 1.1±4.1 | 0.7±1.1 | 0.564 |

Note: LV, left ventricular; RV, right ventricular; RS, radial strain; CS, circumferential strain; LS, longitudinal strain; LAS, left atrial strain; RAS, right atrial strain; RSR, radial strain rate; CSR, circumferential strain rate; LSR, longitudinal strain rate; P1, compared female with male; P2, compare MACE- with MACE+.

# Supplementary Material S7

**Table 7: Sensitivity analysis: Univariate and multivariate Cox regression analysis for prediction of MACE**

| **Outcome** | **Parameters** | **VIF** | **SE** | **HR (95% CI)** | ***p* value** |
| --- | --- | --- | --- | --- | --- |
| Endpoints | Univariable | - | - | - | - |
|  | LGE | 1.037 | 0.365 | 1.795 (0.878-3.667) | 0.109 |
|  | LV GRS | 3.568 | 0.017 | 0.961 (0.929-0.994) | 0.019 |
|  | LV middle RS | 3.535 | 0.018 | 0.961 (0.927-0.996) | 0.030 |
|  | LV diastolic middle CS rate | 3.130 | 0.226 | 0.561 (0.360-0.873) | 0.010 |
|  | LV diastolic apical CS rate | 3.112 | 0.175 | 0.676 (0.480-0.952) | 0.025 |
|  | Multivariable | - | - | - | - |
|  | LV GRS | 1 | 0.016 | 0.966 (0.937-0.996) | 0.026 |
|  | LV diastolic middle CS rate | 1 | 0.260 | 0.528 (0.317-0.879) | 0.014 |
| Hard endpoints | Univariable | - | - | - | - |
|  | LGE | 1.028 | 0.586 | 1.151 (0.365-3.633) | 0.810 |
|  | LV GRS | 3.574 | 0.034 | 0.910 (0.852-0.971) | 0.005 |
|  | LV middle RS | 3.537 | 0.035 | 0.922 (0.861-0.988) | 0.021 |
|  | LV diastolic apical CS rate | 1.020 | 0.215 | 0.498 (0.326-0.758) | 0.001 |
|  | Multivariable | - | - | - | - |
|  | LV GRS | 1 | 0.035 | 0.927 (0.866-0.992) | 0.029 |
|  | LV diastolic apical CS rate | 1 | 0.269 | 0.471 (0.278-0.798) | 0.005 |
| LGE+ endpoints | Univariable | - | - | - | - |
|  | LV GRS | 3.566 | 0.021 | 0.954 (0.916-0.994) | 0.023 |
|  | LV middle RS | 3.566 | 0.022 | 0.956 (0.916-0.997) | 0.037 |
|  | RV middle LS | 1 | 0.029 | 0.942 (0.889-0.998) | 0.041 |
|  | Multivariable | - | - | - | - |
|  | LV GRS | 1 | 0.025 | 0.930 (0.886-0.977) | 0.004 |
|  | RV middle LS | 1 | 0.039 | 0.909 (0.841-0.982) | 0.015 |
| LGE- endpoints | Univariable | - | - | - | - |
|  | LV diastolic middle CS rate | 1.017 | 1.934 | 0.002 (0.001-0.103) | 0.002 |
|  | RV diastolic basal RS rate | 1.017 | 0.054 | 1.141 (1.027-1.268) | 0.014 |
|  | Multivariable | - | - | - | - |
|  | LV diastolic middle CS rate | 1 | 1.934 | 0.002 (0.001-0.103) | 0.002 |
| LGE+ and hard endpoints | Univariable | - | - | - | - |
|  | LV GRS | 3.604 | 0.048 | 0.879 (0.800-0.965) | 0.007 |
|  | LV middle RS | 3.690 | 0.050 | 0.881 (0.798-0.972) | 0.012 |
|  | LV diastolic apical CS rate | 1.046 | 0.247 | 0.512 (0.315-0.830) | 0.007 |
|  | Multivariable | - | - | - | - |
|  | LV GRS | 1.011 | 0.053 | 0.891 (0.803-0.988) | 0.029 |
|  | LV diastolic apical CS rate | 1.011 | 0.319 | 0.450 (0.241-0.841) | 0.012 |

Note: HR, hazard ratio; CI, confidence interval; LGE, late gadolinium enhancement; LV, left ventricular; GRS, global radial strain; RS, radial strain; LS, longitudinal strain; CS, circumferential strain; MACE, major adverse cardiac events; VIF, variance inflation factor.
